# Combating multidrug‐resistant *Plasmodium falciparum* malaria

**DOI:** 10.1111/febs.14127

**Published:** 2017-06-30

**Authors:** Aung Myint Thu, Aung Pyae Phyo, Jordi Landier, Daniel M. Parker, François H. Nosten

**Affiliations:** ^1^ Shoklo Malaria Research Unit Mahidol‐Oxford Tropical Medicine Research Unit Faculty of Tropical Medicine Mahidol University Mae Sot Thailand; ^2^ Centre for Tropical Medicine and Global Health Nuffield Department of Medicine University of Oxford UK

**Keywords:** antimalarial drug resistance, artemisinin resistance, malaria elimination, multidrug resistance malaria, *Plasmodium falciparum*

## Abstract

Over the past 50 years, *Plasmodium falciparum* has developed resistance against all antimalarial drugs used against it: chloroquine, sulphadoxine–pyrimethamine, quinine, piperaquine and mefloquine. More recently, resistance to the artemisinin derivatives and the resulting failure of artemisinin‐based combination therapy (ACT) are threatening all major gains made in malaria control. Each time resistance has developed progressively, with delayed clearance of parasites first emerging only in a few regions, increasing in prevalence and geographic range, and then ultimately resulting in the complete failure of that antimalarial. Drawing from this repeated historical chain of events, this article presents context‐specific approaches for combating drug‐resistant *P. falciparum* malaria. The approaches begin with a context of drug‐sensitive parasites and focus on the prevention of the emergence of drug resistance. Next, the approaches address a scenario in which resistance has emerged and is increasing in prevalence and geographic extent, with interventions focused on disrupting transmission through vector control, early diagnosis and treatment, and the use of new combination therapies. Elimination is also presented as an approach for addressing the imminent failure of all available antimalarials. The final drug resistance context presented is one in which all available antimalarials have failed; leaving only personal protection and the use of new antimalarials (or new combinations of antimalarials) as a viable strategy for dealing with complete resistance. All effective strategies and contexts require a multipronged, holistic approach.

**Abbreviations**
ACT: artemisinin‐based combination therapy
AL: artemether‐lumefantrine
AM: artesunate‐mefloquine
DP: dihydroartemisinin‐piperaquine
EDT: early diagnosis and treatment
FSAT: focal screening and treatment
G6PDd: glucose‐6‐phosphate dehydrogenase deficiency
GMS: greater Mekong Subregions
HRP2: histidine‐rich protein 2 antigen
ITNs: insecticide‐treated nets
LAMP: loop‐amplified isothermal amplification
MDA: mass drug administration
MP: malaria post
MSAT: mass screening and treatment
NMCP: National Malaria Control Program
PQ: primaquine
RDT: rapid diagnostic test
SEA: South East Asia

## Introduction

In 2015, 214 million malaria cases and 438 000 deaths were reported globally, corresponding to an 18% decrease in cases and a 48% decrease in mortality compared to 2000 1. This progress has stimulated efforts to eliminate malaria. The target set by the World Health Organization (WHO) and its partners is to reduce malaria case numbers and the malaria mortality rate globally by at least 90% before 2030 2. However, in the past 10 years *Plasmodium falciparum* isolates with reduced sensitivity to artemisinin and artemisinin derivatives have emerged and spread across South East Asia (SEA), with evidence of their existence stretching from the coast of Vietnam to the Myanmar–India border. There are recent reports of treatment failure in Cambodia, as well as along the Thai‐Myanmar and Vietnam–Cambodia borders, with treatment failing not only for artemisinin alone but also in combination with partner drugs (piperaquine or mefloquine) 3, 4, 5. Further spread of resistance to artemisinin‐based combination therapies (ACTs) could seriously compromise the objective of *P. falciparum* elimination in the Greater Mekong Subregion (GMS). Even more recently, evidence was reported of artemisinin‐resistant strains of *P. falciparum* originating outside of the GMS, in Africa (in a Chinese migrant returning from Equatorial Guinea) 6. An increase in prevalence or geographic dispersal of such strains could result in a public health disaster.

*Plasmodium falciparum* parasites have developed resistance to all antimalarials that have been used against them thus far. Drug resistance in *P. falciparum* has also almost always emerged from Asia, and in particular from Cambodia. In the late 1950s, chloroquine‐resistant parasites emerged from this region and subsequently spread globally 7. Later, pyrimethamine‐resistant parasites from the same region also spread to Africa 8. The emergence of resistance to two life‐saving drugs disastrously led to millions of deaths, especially in sub‐Saharan countries 9.

There are some common factors in these successive histories of emergence and spread of antimalarial drug resistance and it is important to take these factors into consideration with regard to combating drug resistance in *P. falciparum* malaria. Drug resistance is likely to emerge in any situation where there is widespread exposure of parasites to antimalarial 10. For example, the emergence of chloroquine resistance in the 1950s and its subsequent spread globally was worsened by irrational use of the drug. Widespread use of subtherapeutic doses, especially mass dispersal through chloroquine salts, created an optimal environment for the emergence, survival and persistence of chloroquine‐resistant malaria parasites 11, 12. The use of such uncontrolled doses of antimalarial meant that many people had residual circulating levels of the drug. This situation favours selection (or step‐wise selection) of resistant or partially resistant parasites. When it became clear that chloroquine would no longer suffice in treating *P. falciparum* malaria, many regions turned to sulphadoxine‐pyremethamine as the first‐line antimalarial. However, regarding cross‐resistance between drugs, inadequate treatment and poor compliance, the resistance to this combination quickly emerged and spread as well 8, 13.

Given the apparent inevitability of parasites developing resistance to antiparasitic drugs, and the need to treat infected patients, it is important to provide access to treatment in a way that reduces the likelihood of emergence, or slows the spread of resistance. In the past, development of resistance to antimalarials has occurred as a process over time. First, parasites exhibit slightly reduced sensitivity to antimalarials. Then their resistance increases so that either higher doses or longer treatment periods are necessary for parasite clearance. Finally parasites reach a point of full‐blown resistance, where the antimalarial is no longer useful and treatment fails. Given this spectrum of resistance severity, approaches for combating resistance should be tailored to the relevant resistance scenario and malaria control or elimination programmes should take a cautionary stance, with inclusion of a resistance surveillance system alongside malaria epidemiological surveillance. Here, we present an outline for a locally driven drug resistance surveillance system and we present a suite of approaches for combating drug‐resistant *P. falciparum* malaria based on three main scenarios:

- Preventing antimalarial resistance from developing: stewardship of antimalarials
- Dealing with drug‐resistant malaria
- Dealing with complete clinical resistance

## Measurement and surveillance of antimalarial resistance

WHO defines antimalarial drug resistance as the ‘ability of a parasite strain to survive and/or multiply despite the administration and absorption of a drug given in doses equal to or higher than those usually recommended’ 14. This clinical definition is difficult to measure in the field since it requires parasitological monitoring of patients and long duration of follow‐up because of the pharmacokinetics of the drug. In endemic areas, this *in vivo* approach also requires the differentiation of recrudescence from new infections 15. Ideally this type of definition further relies on the measurement of circulating drug levels to ensure that they are adequate.

In the laboratory, *in vitro* assays are used to directly measure the parasite susceptibility to drugs. These assays are well standardized for drugs, such as chloroquine, quinine and mefloquine, and provide a dose–response relationship by measuring the inhibition of growth of parasites in culture, from ring stage to schizonts. Standard drug susceptibility assays are less capable of detecting artemisinin resistance since it affects predominantly ring‐stage parasites. Modified *in vitro* ring‐stage survival assays (RSA) can measure the resistance in an early ring stage, however, they cannot differentiate late ring stages. This occasionally creates inconsistency between *in vivo* and RSA assay results 16.

Finally, progress in sequencing and analysis of genomic data have simplified and accelerated the identification of genetic modifications characteristic of resistant parasites, for example, through genome‐wide association studies 17. The detection of resistance‐related genetic polymorphisms can be used to measure the prevalence of resistant parasites and trends over time (Table 1).

**Table 1:** Associated molecular markers to antimalarial drug resistance

Gene	Variation	Antimalarial	Risk of clinical failure^a^	References
*Pfmdr*	N86Y	Chloroquine	Medium	18
	N86Y	Amodiaquine	High	18
	N86Y	Artemether‐Lumefantrine	Medium	19
	Copy number amplification	Mefloquine	High	20
	Copy number amplification	Mefloquine‐Artesunate	Medium	3, 20
	Copy number amplification	Artemether‐Lumefantrine	High	19
*Pfcrt*	K76T	Chloroquine	High	18
*Pfdhfr*	S108N	Pyrimethamine	Medium	18
	N51I+C59R+S108N	Pyrimethamine	Medium	18
*Pfdhps*	A437G+K540E	Sulphadoxine	Medium	18
*Pfdhfr* + *Pfdhps*	Quintuple	Sulphadoxine‐Pyrimethamine	High	18
*cytochrome b*	Y268S	Atovaquone	High^b^	21
*Kelch‐13*	Position^c^ 210–707	Artemisinins	High	22, 23
*Kelch‐13*	Position^d^ 210–707	DHA‐Piperaquine Mefloquine‐Artesunate	High	3, 24
*Plasmepsin II*	Copy number amplification	DHA‐Piperaquine	High	25, 26
*Chromosome 13*	exo‐E415G	DHA‐Piperaquine	High	26

a Adjusted odds ratio or hazard ratio < 2.0 low risk, ≥ 2 and < 5 medium, ≥ 5.0 high risk.

b Resistance in vitro only.

c Some snps associated with delayed clearance.

d Some snps associated with treatment failures of these acts in the presence of resistance to the partner.

Malaria control and elimination programmes should have epidemiological surveillance systems in place, and antimalarial resistance surveillance should be a component. An antimalarial resistance surveillance system needs to account for both the emergence and spread of resistance, preferably in a spatio‐temporally explicit manner. The system needs to be capable of quickly identifying resistant (or suspected resistant) parasites and reporting their location. It must have both sufficient reservoirs of parasites in order to measure or estimate the population proportion of parasites with reduced sensitivity to antimalarials and must be geographically expansive enough to have an understanding of the spatial dispersion of resistant parasites in the target region.

Surveillance in itself is not enough. The malaria control or elimination programme should have plans for dealing with emerging resistance or the invasion of resistant parasites. Prevention in the first place is ideal.

## Preventing antimalarial resistance from developing: stewardship of antimalarials

Approaches for increasing the time until emergence of resistance include: limiting the inessential use of antimalarials, regulating antimalarial quality, restricting monotherapies and controlling transmission of parasites.

### Limit the inessential use of antimalarial drugs

Resistance to chloroquine emerged during a time when presumptive treatment was widespread but the relatively recent advent of easy‐to‐use and affordable point‐of‐care diagnosis test (rapid diagnostic test or RDT) has made *P. falciparum* malaria diagnosis attainable even in resource poor and remote settings 27. In addition to limiting treatment to confirmed cases, it is important to promote supervised treatment for better compliance to complete the treatment course. Patients who are not monitored may fail to complete a full round of treatment, leading to the exposure of parasites to sublethal doses 28.

### Restrict the use of monotherapy and regulation of antimalarials quality

The use of single antimalarial compounds (monotherapy) can quickly lead to resistance in parasite populations. An alternative approach has been the simultaneous use of multiple compounds, common in treatment of other diseases such as HIV and tuberculosis 29. Combining several (two or more) molecules with different mechanisms of action and different elimination half‐lives may reduce the probability that parasites will develop resistance since resistance to one antimalarial does not always confer resistance to antimalarials of a different class 30, 31.

The provision of quality‐ensured ACT, in which the amount and activity of antimalarial are guaranteed to provide a lethal dose for the parasite, to health facilities is a key element of any strategy for combating drug resistance in malaria. Monitoring the use of antimalarials through official healthcare providers is crucial but has historically been difficult to implement. Even in regions with strong healthcare systems and proactive drug quality assurance components, antimalarials may be procured through the private sector (e.g. through pharmacies or privately owned shops). The multitude of such vendors, and their informal and frequently undocumented status, presents major obstacles not only for treatment of malaria but also with regard to parasite exposure to monotherapies and poor quality antimalarials. Only 20% of WHO state members have well‐established drug regulations 32; clearly there is an enormous need for increased antimalarial quality assurance. Initiatives that replace monotherapy with quality combination therapies are encouraging but need to be scaled up and comprehensive.

### Controlling the dispersal of parasites through reduction of transmission

Disruption of transmission can be achieved by focusing on human hosts, parasites or mosquito vectors. Easy and early access to diagnosis and treatment is crucial not only from medical and prevention standpoints but also as an approach to prevent the transmission of potentially resistant or partially resistant parasites 27. Infections receiving effective treatment within 48 h of developing symptoms are less likely to have developed sufficient sexual stage parasites (gametocytes) for transmission. In *P. falciparum* infections, gametocytes typically appear 5–7 days after the onset of symptoms 33. The inclusion of gametocytocidal drugs to ACT (e.g. primaquine) helps to drastically reduce transmission 34.

Vector‐based approaches are also efficient at reducing transmission, usually either through reducing mosquito vector population sizes or through disrupting contact between humans and mosquitoes. Deployment and use of insecticide‐treated nets (ITNs) or mosquito repellents (individual applications or spatial repellents) can reduce contact between vectors and humans. Use of insecticides (both in treated nets and in residual spraying) can reduce mosquito population numbers and therefore reduce the probability that humans come into contact with mosquito vectors. These approaches have shown success in reducing malaria‐related morbidity and mortality in high transmission settings 35. The use of bed nets in SEA has had mixed results because of diverse mosquito vectors with highly variable blood feeding behaviours 36. Residual spraying may have been effective in reducing malaria morbidity and mortality in some parts of SEA during previous eradication attempts (e.g. in central Thailand) 37. Specific, effective tools for outdoor vector control (personal protection or spraying campaigns) remain to be developed for this region.

Ultimately, holistic approaches are needed for effective interrupting and reducing transmission. One step further from this approach is to push for elimination of malaria from a region under threat of drug resistance.

## Dealing with drug‐resistant malaria

When drug resistance has emerged, it then becomes important to halt its dispersal.

### Increasing access to effective early diagnosis and treatment

Access to diagnosis and treatment is a core intervention of malaria programmes. In a context of increasing drug resistance, it becomes even more crucial to ensure access to continuous, quality early diagnosis and treatment (EDT) in order to terminate transmission foci before resistant parasites become dominant. Networks of community‐based Malaria Post (MP) are being intensively increased in most of GMS countries undergoing malaria elimination. Emphasis must be placed on continuous availability of EDT at community level, through strengthened supervision, uninterrupted supply chains, data and activity monitoring 38. Marginal populations in hard‐to‐reach areas, conflict zones and border areas may not be adequately reached by government services, meaning that nongovernmental organizations can be important players for closing the gaps in public health provision.

### Drug‐related strategies for failing antimalarials and combination therapies

Given the existence of multiple antimalarial combination therapies, one approach to controlling resistance in local parasite populations is to alternate the combination therapy in use. Not only may this delay the onset of resistance to each combination therapy, the development of resistance to one line of antimalarials may also lead to the loss of resistance to another. For example, while mefloquine resistance was widespread in Cambodia, there are recent reports of increased sensitivity to the antimalarial subsequent to the onset of resistance to piperaquine 26. However, this strategy is likely to only work over a short time frame, and can potentially lead to the exhaustion of all possible combinations 39. Other alternatives include alternating combination therapies used in a single community with each patient or increasing the dose or duration of antimalarial regimes 40, 41.

### Aggressive *Plasmodium falciparum* elimination

As parasite populations approach complete resistance to all viable antimalarials, malaria treatment and control options are increasingly limited. One proposed solution for such settings is elimination (rather than control) of the parasite populations. In many low prevalence settings, EDT is likely to be sufficient to eliminate parasites over time 42. However, in emergency situations (with full‐blown resistance nearing), it may be necessary to take more aggressive measures in order to quickly eliminate the parasites from a target region.

In order to reduce parasite populations quickly, elimination must target all individuals who carry parasites, including those undetected and therefore untreated by the health system 43. Asymptomatic carriage was recently described as afflicting a significant proportion of the population even in low transmission areas of SEA 44. Even if each individual only carries low parasite densities, these parasites can persist for long periods of time acting as a reservoir for the next transmission season 45.

**Figure 1:**
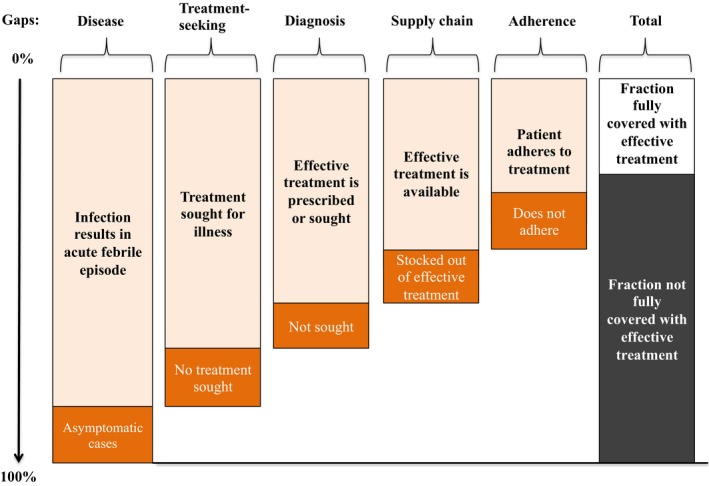
Potential coverage gaps that determine the fraction of infections rapidly identified and treated. Figure reproduced from 46.

The backbone of an aggressive elimination approach is identical to enhanced control interventions and consists of reducing as much as possible the number of clinical episodes left untreated through increased access to malaria EDT (Fig. 1). In addition to case management, additional interventions need to be integrated to address the asymptomatic reservoir: detection and treatment of asymptomatic‐infected individuals or targeted treatment to the transmission source 43.

The ‘screen and treat’ strategies are largely dependent on the sensitivity of the screening method 47. Reactive Case Detection (RCD) and Focal Screening and Treatment (FSAT) strategies consist of screening clinical cases around an index case. These approaches assume that spatio‐temporal clustering is strong and predictable, which may not be the case given complex transmission patterns in some areas 48, 49. At a broader scale, Mass Screening and Treatment (MSAT) can be used to target an index community in a similar manner. In presumptive treatment strategies, drugs are administered to all individuals within a target population (house, village, etc.), which overcomes the hurdle of detection thresholds. Targeted mass drug administration (MDA), for example, targets high prevalence communities and aims to interrupt transmission by draining the human reservoir of parasites. MDA interventions can be triggered after a survey providing a direct estimate of the prevalence of malaria. Identification of submicroscopic carriers requires more sensitive methods than RDT or microscopy, such as ultrasensitive molecular techniques 50. The current RDT cannot detect parasitaemia below 100 parasites·μL^−1^ for *P. falciparum* and this would leave more than 75% of infected individuals undetected in SEA contexts 50. A new generation of more sensitive field tests is under development and undergoing field trials: hypersensitive RDT detecting lower concentrations of histidine‐rich protein 2 (HRP2) antigen, loop‐amplified isothermal amplification (LAMP) 51, or new serological tests detecting exposure to Plasmodium parasites. Significant gains can be expected for elimination interventions if these tests can improve the proportion of carriers successfully detected (for RCD, FSAT, MSAT) or simplify the logistics and reduce the costs of hotspot identification (for MDA).

Criteria for targeting specific thresholds of asymptomatic prevalence are to be defined, and may vary depending on the transmission intensity (entomological parameters, exposure). A crucial factor in the success or failure of MDA to interrupt transmission is population coverage (for rapid and widespread parasite clearance) and the continuous functioning MP (to detect and treat any reimported infections).

The inclusion of a malaria vaccine could also be helpful for achieving malaria elimination. The most advanced vaccine is RTS ASO1, and it could be the first to be licensed. The vaccine alone is unlikely sufficient for achieving elimination because of its limited effectiveness 52. However, if used in combination with EDT, MDA and with vector‐based strategies, it may prove effective as a tool for targeted elimination.

## Dealing with complete clinical resistance

Once treatment with extant antimalarials or combination therapies is no longer viable, options become limited and focus either on halting transmission or on the advent of new antimalarials or new combinations of antimalarials. When treatment is no longer possible, protection from exposure to infectious mosquito vectors is of heightened importance. Viable vector‐based strategies include methods that decrease vector population sizes (residual spraying), repelling vectors from human habitats (spatial repellents) and personal protective approaches, such as treated clothing, bed nets and insect repellents applied as ointment. Residual transmission would remain a problem. Furthermore, infected persons with gametocytes in their blood may be untreatable from a clinical perspective, but their importance in transmission must be considered. Transmission blocking drugs (primaquine) would still carry importance as would the use of ITNs in order to disrupt onward transmission.

Antimalarial‐based approaches to complete drug failure are currently limited. Nearly all antimalarials belong to three families or classes of chemical compounds: quinolones, antifolates and peroxides. Current treatment options for nonsevere cases rely on four ACTs in which an artemisinin derivative is partnered with lumefantrine, piperaquine, mefloquine or amodiaquine (Table 2). Antimalarials with similar structures and/or similar mechanisms of action are prone to cross resistance 53, 54.

**Table 2:** WHO recommended first‐line antimalarial drugs

WHO approved first‐line antimalarial drugs for uncomplicated malaria	Artemether + Lumefantrine
	Artesunate + Amodiaquine
	Artesunate + Mefloquine
	Dihydroartemisinin + Piperaquine
	Artesunate + Sulfadoxine‐Pyrimethamine (SP)

New therapeutic strategies are critically needed to eliminate multidrug‐resistant malaria. One option currently being tested is the inclusion of an additional partner drug to existing combinations: triple combination therapy, with combinations such as artesunate‐lumefantrine‐amodiaquine and dihydroartemisinin‐piperaquine‐mefloquine (ClinicalTrials.gov Identifier:NCT02453308) currently undergoing phase 3 trials. As in HIV and tuberculosis, relying on additional drugs creates additional barriers to resistance, theoretically decreasing chances that parasites will acquire the necessary mutation or mutations that confer resistance to each of the drug cocktail components 55. However, it is possible that parasites will develop resistance mechanisms that are effective in evading all antimalarial compounds included in the combination, perhaps especially in a context where there are circulating parasites that have already developed resistance to several compounds. Triple combinations are expected to provide some relief against multidrug‐resistant malaria in regions such as Western Cambodia, but they may quickly fall to resistance. Increased toxicity (among human hosts) from multiplying the number of active compounds is also a concern.

The other major alternative is the introduction of new antimalarial compounds. Three new compounds show potential to substitute current artemisinin derivatives and they are being tested in ongoing phase 2 studies: OZ439 or artefenomel (trioxalene) KAF156 (imidazolopiperazine class) and KAE 609 (spiroindolone class) all possess rapid parasite clearance half‐lives on both wild‐type parasites and parasites exhibiting K13 mutations (which are related to artemisinin resistance) 56, 57. No serious drug‐related adverse effects have been reported for these compounds. Their elimination half‐lives are significantly longer than that of artemisinin derivatives. Another three new promising antimalarials will soon begin phase 2 studies: AQ‐13 (aminoquinoline class with modified aliphatic side chain), ferroquine (ferrocene–quinolone conjugate) and DSM‐265 (inhibitor of dihydroorotate enzyme). Their action on parasites is slower than artemisinin derivatives but they have elimination half‐lives of 14 days, 16 days and 4 days respectively 57, 58. New drugs are urgently needed, however, it will be years before any are ready to be rolled out as first‐line treatments for *P. falciparum* malaria.

## Conclusion

While sub‐Saharan Africa has by far the heaviest burden of *P. falciparum* morbidity and mortality, SEA has historically played an important role with regard to drug‐resistant *P. falciparum*. Previous strains of resistant parasites that are likely to have emerged in SEA have subsequently dispersed to Africa, resulting in devastating loss of life. The world was slow to react to this disaster and to replace failing drugs with more effective artemisinin‐based combinations 59, 60. The deployment of these treatments largely contributed to a reduction in the number of cases, particularly in SEA. However, these gains are now threatened by the emergence of resistance to the artemisinin. Containment efforts failed and parasite strains from several independent origins have spread to the entire SEA subcontinent. The impact of these changes in the genetics of the parasite population is barely visible outside the laboratories, because the partner drugs continue to clear most infections presenting in the clinics. However, in Cambodia and on the Thai‐Myanmar border, where some alleles (e.g. C580Y) appear to be moving toward fixation, the partner drugs (piperaquine and mefloquine respectively) are now also falling to resistance, paving the way for a new disaster. Since new antimalarials are still years away, there is a race against time to rapidly eliminate *P. falciparum* from the region while some ACTs retain efficacy. This is achievable with existing tools but requires a strong political commitment and financial support. Like in any war, combating multidrug‐resistant *P. falciparum* requires good intelligence and prompt reaction. Modern information technology provide the means to detect, enumerate, map and monitor cases of malaria, resistance and foci of transmission. Early detection and treatment of clinical cases, detection of submicroscopic reservoirs and adapted vector control are the three pillars of a successful elimination. The cost of failing to eliminate artemisinin‐resistant *P. falciparum* from SEA and its spread to Africa is simply unaffordable.

## Author contributions

AMT wrote the first draft. APP, JL, DP and FN provided critical feedback, comments and revisions for the final manuscript. All authors have read and approved the final version of manuscript.

## Conflicts of interest

All authors have declared no competing interest. The authors are currently involved in *Plasmodium falciparum* elimination efforts in Eastern Kayin State, Myanmar. This may influence their opinions about elimination strategies.
